# Melatonin and cancer risk: does light at night compromise physiologic cancer protection by lowering serum melatonin levels?

**DOI:** 10.1038/sj.bjc.6601626

**Published:** 2004-03-02

**Authors:** E S Schernhammer, K Schulmeister

**Affiliations:** 1 Channing Laboratory, Department of Medicine, Brigham and Women's Hospital and Harvard Medical School, 181 Longwood Avenue, Boston, MA 02115, USA; 2 Ludwig Boltzmann-Institute for Applied Cancer Research, KFJ-Spital, Vienna, Austria; 3 ARC Seibersdorf research, Health Physics Division, Seibersdorf A-2444, Austria

**Keywords:** melatonin, cancer, light exposure, night work

## Abstract

The suprachiasmatic nuclei in the hypothalamus, one of the most important physiological determinants of alertness and performance, drive a circadian pacemaker in mammals, with an intrinsic period averaging 24 h. Light is the primary stimulus to the disruption and resetting of this pacemaker, which is expressed in changing melatonin rhythms. Melatonin production in humans decreases when people are exposed to light at night. Since melatonin shows potential oncostatic action in a variety of tumours, it is possible that lowered serum melatonin levels caused by exposure to light at night enhance the general tumour development. Cancer is the second leading cause of death in industrialised countries like the United States, where a significant proportion of workers engage in shift work, making a hypothesised relation between light exposure at night and cancer risk relevant. Observational studies support an association between night work and cancer risk. We hypothesise that the potential primary culprit for this observed association is the lack of melatonin, a cancer-protective agent whose production is severely diminished in people exposed to light at night.

## 

### Light and melatonin

Light at night is an environmental exposure that has become integral to our lives. Before 1879, when the light-bulb was invented, humans were exposed to only insignificant amounts of low-intensity light at night from sources such as candles and petroleum lamps. Later on, paralleled by growing industrialisation, a need for longer days emerged, with electric light making it possible to push multifaceted working schedules further and further into the night. Today, exposure to light at night, both in the form of occupational exposure during night work and as a personal choice and lifestyle, is experienced by numerous night-active members of our societies. We, however, do not appear to be well adapted to bright nights: shift workers, for instance, reportedly suffer from a variety of health problems (Vener *et al*, 1989; Costa, 1997; Boggild and Knutsson, 1999). Previously, humans tended to conduct their daily activities according to the sun's cycle: rising at sunrise and going to bed at sunset. Such sleep rhythms appear not only to be more natural, but also to be essential for a variety of physiologic functions in humans, such as body temperature, excretion, and the production of hormones (Weitzman *et al*, 1981; Czeisler and Klerman, 1999). Melatonin, for example, follows a very distinct pattern of production, which is very closely linked to the individual's circadian rhythm, following light exposure: during the day, almost no melatonin is produced, whereas during the night, when it is dark, almost all melatonin is produced (Snyder *et al*, 1967). Environmental lighting powerfully alters physiologic release of melatonin, which typically peaks in the middle of the night (Zeitzer *et al*, 2000): a profound melatonin reduction was observed in humans after 2 weeks of intermittent nightly exposure to light (Zeitzer *et al*, 2000; Graham and Cook, 2001). While levels may recover during the periods of sleep, they will not recover to their maximal amount unless habits are kept constant, because any disruption of a natural circadian rhythm will diminish the ability to recover from that suppression.

### Link between light at night and breast cancer risk through the oestrogen pathway

Past studies suggested that the excess breast cancer mortality seen in urban areas as well as the North East as compared to the South of the US could be due to differences in exposure to sunlight (Garland *et al*, 1990), perhaps mediated through the vitamin D pathway. However, based on a large body of experimental work in the 1960s and 70s, evidence grew that visible light, including artificial light, can acutely suppress melatonin. Thus, novel hypotheses were generated, proposing that the diminished function of the pineal gland might promote the development of breast cancer in humans. One of the initial theories supporting that a diminished function of the pineal gland might promote the development of cancer hypothesised that melatonin suppression may lead to an increase in levels of reproductive hormones, particularly oestradiol, thereby increasing the growth and proliferation of hormone-sensitive cells in the breast (Cohen *et al*, 1978; Stevens, 1987).

Observational studies have supported that theory, indicating that women in occupations that expose them to light at night do experience a higher risk of breast cancer, and that blind women, who do not have the ability to experience lower melatonin levels because of their supposed lack of receptivity to light, have a lower incidence of breast cancer (Schernhammer and Hankinson, 2003). Studies fairly consistently report meaningful increases in breast cancer risk among postmenopausal women exposed to shift work (Pukkala *et al*, 1995; Tynes *et al*, 1996; Davis *et al*, 2001; Hansen, 2001; Rafnsson *et al*, 2001; Schernhammer *et al*, 2001). Two retrospective studies of flight attendants with occupational exposure to light at night linked the employment time to an increased risk of breast cancer (Pukkala *et al*, 1995; Rafnsson *et al*, 2001). Two nationwide record linkage studies (Tynes *et al*, 1996; Hansen, 2001) and a retrospective case–control study (Davis *et al*, 2001) associated night work with an approximately 50% higher risk of breast cancer (Tynes *et al*, 1996; Hansen, 2001). Finally, the Nurses’ Health Study, the only prospective study published that evaluated the association, observed a positive association of extended periods of rotating night work and breast cancer risk (more than 30 years of rotating night work: RR=1.36; 95% CI=1.04–1.78) (Schernhammer *et al*, 2001). In this study, night work was defined as the total number of years during which the nurses had worked rotating night shifts with at least three nights per month, in addition to days and evenings in that month. During 10 years of follow-up, 2441 incident cases of breast cancer were documented among 78 562 women. A positive association between the number of years a woman had worked on rotating night shifts and breast cancer risk was observed (test for trend, *P*=0.02). Among postmenopausal women, the relative risk for breast cancer, controlling for all the major risk factors for breast cancer, was moderately increased after 1–14 and 15–29 years of rotating night shift work, and was further increased (RR 1.36; 95% CI 1.04–1.78) for those nurses who worked the night shift for 30 or more years, with similar risks for premenopausal women (RR 1.34; 95% CI 0.77–2.33). Thus, in sum, observational studies seem to support the hypothesis that night work increases the risk for breast cancer. This association may be mediated, at least in part, by the oestrogen pathway.

### Light at night and other cancers

Only few previous observational studies have addressed the relationship between shift work and cancers, other than breast cancer. Early suggestions for an increased cancer risk related to shift work arose from two mortality studies that were conducted among male shift workers to assess the influence of shift work upon total and cause-specific mortality, with suggestions for an increased cancer mortality related to shift work. Tynes *et al* report an increased risk of colon (SIR 1.3; 95% CI 0.6–2.6) and rectum cancer (SIR 1.8; 95% CI 0.7–3.9) in their cohort of female radio and telegraph workers. Rafnsson and colleagues do not report the risks for colorectal cancer among the female Icelandic flight attendants, but describe an elevated risk for tumours of the lymphatic system.

## CANCER-PROTECTIVE EFFECTS OF MELATONIN

In recent years, an overwhelming amount of research has been devoted to exploring the cancer-protective properties of the hormone melatonin. Today, many of the oncostatic properties of melatonin have been fairly well described (Vijayalaxmi *et al*, 2002), and evidence from experimental studies strongly suggests a link between melatonin and tumour suppression (Schernhammer and Hankinson, 2003). *In vitro* studies, although not entirely consistent (Panzer *et al*, 1998), give support to a reduction in the growth of malignant cells of the breast (Hill and Blask, 1988; Cos *et al*, 1996, 1998, 2002; Mediavilla *et al*, 1999) and other tumour sites (Sze *et al*, 1993; Ying *et al*, 1993; Petranka *et al*, 1999; Shiu *et al*, 1999; Kanishi *et al*, 2000) by both pharmacological and physiologic doses of melatonin. In rodent models, pinealectomy boosts tumour growth (Tamarkin *et al*, 1981), whereas exogenous melatonin administration exerts anti-initiating (Musatov *et al*, 1999) and oncostatic activity (Anisimov *et al*, 1997, 1999; Cini *et al*, 1998; Mocchegiani *et al*, 1999) in various chemically induced cancers. The most prominent mechanisms proposed to explain the oncostatic action of melatonin include its antimitotic and antioxidant activity (Brzezinski, 1997), and its potential modulation of cell-cycle length through control of the p53–p21 pathway (Mediavilla *et al*, 1999). Melatonin is believed to have antimitotic activity by its direct effect on hormone-dependent proliferation through interaction with nuclear receptors. Another explanation is that melatonin increases the expression of the tumour-suppressor gene p53. Cells lacking p53 have been shown to be genetically unstable and thus more prone to tumours.

*In vitro* studies do support not only an effect of melatonin on breast cancer (Hill and Blask, 1988; Cos *et al*, 1996, 1998, 2002; Mediavilla *et al*, 1999), but also on other tumours. In fact, to date, melatonin has been shown to be oncostatic for a variety of tumour cells in experimental carcinogenesis (Sze *et al*, 1993; Ying *et al*, 1993; Petranka *et al*, 1999; Shiu *et al*, 1999; Kanishi *et al*, 2000). Reports show that melatonin exhibits a growth-inhibitory effect on endometrial (Kanishi *et al*, 2000) and ovarian carcinoma cell lines (Petranka *et al*, 1999), Lewis lung carcinoma (Mocchegiani *et al*, 1999), prostate tumour cells (Laufer *et al*, 1999), and intestinal tumours (Anisimov *et al*, 1997, 2000a, 2000b), for example. Furthermore, today, several clinical trials confirm the potential of melatonin, either alone or in combination with standard therapy regimens, to generate a favourable response in the treatment of human cancers (Vijayalaxmi *et al*, 2002).

## UPDATING THE MELATONIN HYPOTHESIS

Given the evidence from experimental studies supporting the general oncostatic property of melatonin, we therefore speculate that exposure to light at night not only has an impact on breast cancer risk, but also may increase the risk of other cancers, primarily through the melatonin pathway. This has been posed previously (Kerenyi *et al*, 1990) without much further attention from the scientific community, but most recent evidence from observational studies supports such a link. The Nurses’ Health Study Cohort was used to explore the association between night work and colorectal cancer; 602 women were diagnosed with incident colorectal cancer during the 10 years of follow-up. In these analyses, women who worked 15 or more years on rotating night shifts were at a higher risk of colorectal cancer than were women who never worked rotating night shifts. The relative risks after adjustment for age, smoking, physical activity, and other colorectal cancer risk factors were 1.00 (0.84–1.19) for 1–14 years on rotating night shifts and 1.35 (1.03–1.77) for 15 or more years on rotating night shifts (test for trend, *P*=0.04).

On the basis of the observed effect of night work on colorectal cancer, which, contrary to breast cancer cannot be fully explained by variations in circulating oestrogen levels, taken together with consistent evidence from experimental studies, we believe that the effects of light exposure at night on cancer risk may be explained, at least in part, by the direct oncostatic mechanisms of melatonin. We propose that an increase in light exposure during the night decreases the amount of time available for melatonin production, which reduces the possible nonspecific oncostatic effect of the pineal gland, thus increasing the risk not only of breast cancer but also of other cancers. Although other aetiologic mechanisms, such as the loss of normal diurnal variation in cortisol (Spiegel and Sephton, 2002), may be involved in the influence of night work on cancer risk, we suggest that melatonin may be the primary culprit for this association. Future research should therefore evaluate the relationship of light exposure to the risk of not only breast but also other cancers, including cancer risks in men. There is also a need for research that addresses individual susceptibility and the question whether individuals with lower melatonin levels choose to do night work more often than do those with higher melatonin levels. First and foremost, however, associations between melatonin levels and cancer risk in humans need to be evaluated.
